# Sex Differences and Effects of Predictive Cues on Delayed Punishment Discounting

**DOI:** 10.1523/ENEURO.0225-19.2019

**Published:** 2019-08-19

**Authors:** Anna E. Liley, Daniel B. K. Gabriel, Helen J. Sable, Nicholas W. Simon

**Affiliations:** Department of Psychology, University of Memphis, Memphis, Tennessee 38152

**Keywords:** cues, decision-making, delay discounting, estrous, punishment, sex differences

## Abstract

The majority of the research studying punishment has focused on an aversive stimulus delivered immediately after an action. However, in real-world decision-making, negative consequences often occur long after a decision has been made. This can engender myopic decisions that fail to appropriately respond to consequences. Whereas discounting of delayed rewards has been well studied in both human and animal models, systematic discounting of delayed consequences remains largely unexplored. To address this gap in the literature, we developed the delayed punishment decision-making task. Rats chose between a small, single-pellet reinforcer and a large, three-pellet reinforcer accompanied by a mild foot shock. The shock was preceded by a delay, which systematically increased throughout the session (0, 4, 8, 12, 16 s). On average, rats discounted the negative value of delayed punishment, as indicated by increased choice of the large, punished reward as the delay preceding the shock lengthened. Female rats discounted delayed punishment less than males, and this behavior was not influenced by estrous cycling. The addition of a cue light significantly decreased the undervaluation of delayed consequences for both sexes. Finally, there was no correlation between the discounting of delayed punishments and a traditional reward delay discounting task for either sex. These data indicate that the ability of punishment to regulate decision-making is attenuated when punishment occurs later in time. This task provides an avenue for exploration of the neural circuitry underlying the devaluation of delayed punishment and may assist in developing treatments for substance use disorders.

## Significance Statement

Consequences that occur long after a decision are often undervalued, particularly in substance use disorders. However, “delayed punishment discounting” is widely understudied in decision neuroscience. Here, we present a novel delayed punishment decision-making task in rats that reveals robust sex differences, demonstrates the efficacy of punishment predictive cues for reducing punishment discounting, and shows that punishment discounting is independent of reward-discounting. This provides critical information regarding a cognitive process that can contribute to maladaptive decision-making.

## Introduction

Punishment describes the relationship between an action and a resultant aversive outcome (Simon et al., 2009; Jean-Richard-Dit-Bressel et al., 2018). The majority of the research studying punishment has focused on consequences received immediately after an action; however, punishment often occurs long after a decision has been made. For example, an individual that spends their entire paycheck at a bar may not experience immediate consequences but will face eviction weeks later for being unable to pay rent. Preceding consequences with a delay evokes undervaluation of the impending punishment, diminishing its influence over behavior (Yates and Watts, 1975; Strathman et al., 1994; Murphy et al., 2001; Rodríguez et al., 2018). We label this time-evoked transformation in punishment valuation as “delayed punishment discounting”. Understanding this construct is fundamental for addiction neuroscience, as consequences often manifest long after the onset of drug seeking.

Previous work has shown that humans discount costs as a function of delay, and that the magnitude of discounting is distinct from delayed reward discounting (Murphy et al., 2001). Early research reported that animals favor postponed foot shocks relative to prompt foot shocks (Deluty et al., 1983). Rodríguez et al. (2018) began to address delayed punishment discounting during economic decision-making in rats, observing that rats preferred a smaller reward as a function of delay compared with a large, punished reward during ascending and descending delays. In rhesus monkeys, aversive histamine injections weakened cocaine self-administration, an effect attenuated by incremental delays preceding histamine (Woolverton et al., 2012). To further investigate sensitivity to delayed consequences in rats, we developed a two-choice behavioral paradigm called the Delayed Punishment Decision-making Task (DPDT). Rats were trained to choose between a single-pellet reinforcer and a three-pellet reinforcer accompanied by a mild foot shock (0.35 mA). As the task progressed, a delay was introduced preceding the shock that was systematically increased throughout the session (0, 4, 8, 12, 16 s), followed by a final block in which the shock was no longer present.

Male and female rats respond differently to both punishment and non-contingent aversive stimuli (Gruene et al., 2015; Orsini et al., 2016; Jean-Richard-Dit-Bressel et al., 2018). A subset of females display heightened locomotion when anticipating foot shock, whereas males more consistently demonstrate attenuated locomotion (Archer, 1975; Gruene et al., 2015). Chowdhury et al. (2019) observed that female rats are more sensitive to probabilistic punishment during reward seeking than males. During economic decision-making, female rats choose rewards associated with risk of punishment less than males, and omit trials associated with risk more than males (Orsini et al., 2016). Currently, little is known about sex differences in sensitivity to delayed punishment during decision-making. To address this, we compared males and females in the DPDT, and examined effects of estrous cycle on DPDT performance in female rats.

Rewards and punishment are regularly associated with environmental cues, which enable outcome-specific representations that bias decision-making toward positive outcomes and away from aversive outcomes (Barberini et al., 2012). Cues predicting delayed rewards have been shown to affect delay discounting acquisition and sensitivity to pharmacological and neuronal manipulations (Cardinal et al., 2000; Zeeb et al., 2010). Cues can also serve as “conditioned punishers”, signaling impending punishment and driving avoidance behavior in the absence of punishing stimuli (Oleson et al., 2012). These cues may increase the salience of impending punishment, thus reducing punishment discounting. We tested this by inserting a punishment-predictive cue that bridged the gap between action and consequences in the DPDT, then measuring sensitivity to delayed consequences in male and female rats.

Assessment of the neuronal and pharmacological correlates of discounting rewards as a function of delay is a fundamental area of interest in decision neuroscience (Ballard and Knutson, 2009; Roesch and Bryden, 2011; Simon et al., 2013). However, it remains unclear whether the value transformation of delayed rewards and delayed punishment share a common mechanism. To address this on a behavioral level, we compared performance in the DPDT with reward preference in a version of the traditional delay discounting task.

The current experiments addressed four fundamental research questions about the discounting of delayed punishment: (1) do rats discount the negative value of delayed punishment as a function of delay during decision-making, (2) are there sex differences in sensitivity to delayed punishment, (3) do cues bridging actions and consequences alter delayed punishment discounting, and (4) is there a relationship between the discounting of delayed punishments versus delayed rewards?

## Materials and Methods

### Subjects

Ten male and 10 female Long–Evans rats (*n* = 20, Envigo) aged ∼70 d were pair-housed, with some individually housed in cases of excessive aggression or food domination. This cohort of rats was used for all experiments described in this manuscript. Rats were kept on a reversed 12 h light/dark cycle (lights off 8:00 A.M. to 8:00 P.M.), with all procedures conducted during the dark cycle to maximize activity. During behavioral testing, rats were maintained at 85% of their free-feeding weight (with allowances for growth) and had *ad libitum* access to water. All animal procedures were approved by the University of Memphis Institutional Animal Care and Use Committee Animal Care and Use Committee.

### Apparatus

Testing was conducted in standard rat behavioral test chambers (Med Associates) housed within sound attenuating cubicles. Each chamber was equipped with a recessed food pellet delivery trough fitted with a photo beam to detect head entries, and a 1.12 W lamp to illuminate the food trough. Food pellets were delivered into the food trough, 2 cm above the floor centered in the side wall. Two retractable levers were located on the left and right side of the food trough, 11 cm above the floor, with cue lights located directly above each lever. A 1.12 W house light was mounted on the opposing side wall of the chamber. Beneath the house light was a circular nose-poke port equipped with a light and photo beam to detect entry. The floor of the test chamber was composed of steel rods connected to a shock generator that delivered scrambled foot shocks. Locomotor activity was assessed throughout each session with infrared activity monitors located on either side of the chamber just above the floor. Test chambers were interfaced with a computer running custom-written codes through MedPC software (Med Associates), which controlled all external cues and behavioral events.

### The delayed punishment decision-making task

The delayed punishment task measured the influence of punishment on reward magnitude-based decision-making. In brief, rats chose between one and three food pellet reinforcers, with the larger option accompanied by a foot shock that occurred systematically later in time as the task progressed. Sessions consisted of 6 blocks with 12 trials each. Each trial began with illumination of the house light and food trough, after which rats were required to nose poke into the lit trough within a 10 s period to initiate the trial (failure to initiate resulted in the trial being scored as an omission). A nose poke extinguished the trough light, and then caused either a single lever or two levers on both sides of the trough to extend. The first two trials of each block were forced choice trials, with only a single lever available to establish the reward/punishment parameters of each lever individually within the current block. After forced choice trials, the following 10 trials were free-choice trials in which both levers extended simultaneously, allowing rats to choose a preferred lever/reinforcement schedule.

**Figure 1.**
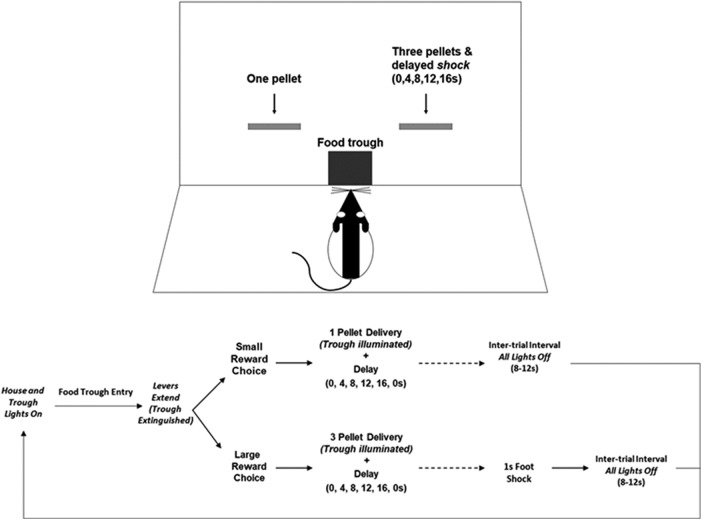
Diagram of the DPDT. After initiating the trail with a trough entry, rats chose between two levers, one lever delivering one pellet reward and the other delivering three pellets accompanied by a delayed foot shock (0, 4, 8, 12, 16 s). There was no shock associated with reward in the final block. Lever identity was counterbalanced across subjects.

The choice of one lever resulted in immediate delivery of a single pellet, and the other caused immediate delivery of three pellets (spaced out over a 3 s period), in addition to a mild foot shock (0.35 mA). Identity of levers (left vs right) was fixed across all sessions and counterbalanced between subjects. During the first block, the shock occurred immediately after lever press; subsequent blocks introduced a delay preceding shock that was progressively extended to 4, 8, 12, and 16 s across blocks. If the unpunished lever was chosen, the intertrial interval (ITI) was increased by a period equivalent to the delay preceding shock (4, 8, 12, or 16 s) to maintain consistency of trial length regardless of choice. After food delivery, delay, and shock (when large reward was chosen), the house light extinguished and an ITI of 10 ± 2 s preceded the next trial. Figure 1 displays the progression of a single DPDT free-choice trial. If rats did not engage an extended lever within the allotted 10 s, the trial was scored as an omission and followed by the ITI. After completion of all five blocks, rats performed a sixth block in which the large reward was no longer accompanied by a foot shock to confirm a preference for the large reward in the absence of punishment.

Foot shock amplitude began at 0.1 mA and was increased by 0.05 mA in the following session if rats completed >85% of trails. This incremental increase in shock intensity limited omissions and allowed all rats to acquire task parameters. On reaching the final shock intensity of 0.35 mA, subjects trained for a minimum of 20 consecutive sessions, or until stable choice performance was achieved, defined as no significance in a repeated-measures day by block ANOVA over the final 5 d of behavior. After rats reached stability, decision-making was compared between male and female rats.

### Sex differences in decision-making

After all rats achieved stability, we compared males and females in the discounting of delayed punishment. In addition, we observed any variations in response suppression and fear expression by analyzing locomotor activity and response omissions for both male and female rats during the DPDT. To test whether fluctuations in estrogen are involved with discounting of delayed punishment during decision-making, we measured the estrous cycle of all female rats after behavioral testing. The ovulation cycle for female rats occurs every 4–5 s and consists of four phases: pro-estrus (12–14 h), estrus (25– 27 h), metestrus (6–8 h), and di-estrus phase (55–57 h; Westwood, 2008). We tested the relationship between ovulation and DPDT by comparing discounting of delayed punishment within female subjects across all four phases of the estrous cycle.

### Vaginal lavage procedures

Rats were vaginally lavaged using autoclaved mini pipettes and Barnstead water immediately after each test session for a 2 week period once stable behavior was reached during the task. Samples were placed onto microscope slides, allowed to dry for 24 h, stained using cresyl violet acetate solution (Nissl staining), and observed under a light microscope to determine cell types. Identification criteria for the four stages of the estrous cycle included: (1) pro-estrus: cells were nucleated and had a granular appearance, (2) estrus: cells were cornified (rounded, with jagged edges), (3) metestrus: cells contained both cornified cells and leukocytes, and (4) di-estrus: cells were leukocytes with some nucleated cells (Goldman et al., 2007; Orsini et al., 2016). If an estrous phase occurred on more than one session, behavioral data for that phase was averaged together during data analysis (Orsini et al., 2016).

### Cue control over punishment discounting

To test the effects of punishment-predictive cues on the discounting of delayed punishment, rats trained for an additional 10 d on a cued version of DPDT. This task was identical to the initial DPDT, except succeeding the selection of the punished lever during the task, a cue light was activated. The cues were located directly above the retractable levers; for each rat, the cue presented was congruent with the lever associated with large reward/delayed punishment (left/right location of this lever was counterbalanced across subjects). This cue remained illuminated until the foot shock was delivered, thus serving as a conditioned punishment signal that bridged the gap between the lever press and delayed foot shock (4, 8, 12, 16 s). During trials in which the punishment was delivered immediately, no cue was presented.

### Contrasting delayed punishment with delayed reward

Measuring the discounting of delayed rewards, or impulsive choice, was similar to procedures described earlier (Simon et al., 2010). In brief, sessions consisted of five blocks with 12 trials each. Each block commenced with two forced choice trials (1 for each lever), followed by 10 free-choice trials. Rats chose between a small, immediate reward (1 pellet) and a large reward (3 pellets) delivered after a delay that increased (0, 4, 8, 12, 16 s) with each block. As with DPDT, identity of levers was counterbalanced across groups. Percentage choice of the delayed reward was used as a measure of impulsive choice, with lower preference for the delayed reward indicative of elevated impulsivity (Orsini et al., 2017). Rats ran on this task until they reached stable performance (30 d).

### Experimental design and statistical analyses

All behavioral data were compiled using custom-made MATLAB scripts, and all statistical analyses were conducted using IBM SPSS Statistics 24. Any violations of Mauchly’s test of sphericity were taken into account and statistical reporting was adjusted by reporting Greenhouse–Geisser values, with degrees of freedom adjusted accordingly. Stable decision-making in either the DPDT or delay discounting task was tested using a day × block ANOVA across the final 5 d of testing and was defined as follows: (1) lack of main effect of day, and (2) lack of significant day by block interaction. The average percentage choice of punished reward across these 5 d of stability as well as the slope of percentage choice of the punished reward from blocks 1 through 5 were calculated as complementary measures of delayed punishment discounting. Sex differences were assessed using a sex × block mixed ANOVA. Furthermore, data collected from females from each phase of the estrous cycle (proestrus, estrus, metestrus, and diestrus) were compared using a phase × block ANOVA.

Locomotion was used as an indirect measure of expectation of delayed punishment during the DPDT. We compared locomotion during the delay preceding foot shock with locomotion during the matched delay after small reward delivery on punishment-free trials (Fig. 1). Locomotion for punished and safe levers was measured as total percentage of time spent moving, was averaged across all delay lengths (individual blocks could not be analyzed because some rats never chose the punished reward with certain delays), then compared using a paired samples *t* test. Data from three female rats were removed from locomotion analysis because of avoidance of the punished lever. The effects of conditioned cues on behavior were measured using a task (cued versus uncued) × block repeated-measures ANOVA. To compare the discounting of delayed punishment with delayed reward, a bivariate correlation was run to compare (1) slope of large reward preference across all blocks for both DPDT and delay discounting tasks, and (2) area under the curve (AUC) across all blocks for each task. Finally, we compared the rate of discounting between reward and punishment by comparing the absolute value of the slopes between tasks using a two-way mixed sex × task ANOVA. Absolute value was used to control for the direction of the curve, as DPDT produces an upward curve, whereas delay discounting produces a downward curve.

## Results

### DPDT

**Figure 2.**
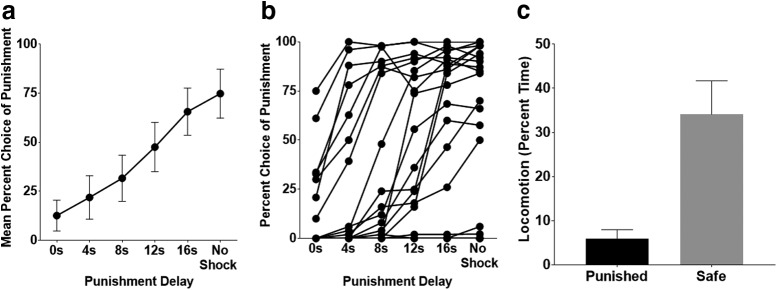
DPDT. ***a***, On average, rats shifted behavior toward the punished reward as the delay increased, indicative of underestimation of delayed punishment. Each marker represents mean choice of the large reward ± SEM. ***b***, Individual differences in DPDT performance, with each line representing a single subject. ***c***, Percentage time spent performing locomotion during the delay period after punished and safe levers were pressed and reward was delivered. Locomotion significantly decreased after choice of the punished relative to safe reward, suggesting that rats anticipated impending shock.

Female rats required more sessions than males to achieve stable responding (*t*_(20)_ = 5.243, *p* < 0.001; female mean: 32.2 sessions, male mean: 28 sessions). After rats achieved stability, a repeated-measures ANOVA of the 5 d average revealed a significant effect of punishment delay (*F*_(2.269,36.306)_ = 17.766, *p* < 0.001), such that rats selected the punished reward more frequently during blocks in which punishment was delayed, with this preference increasing during longer delays (Fig. 2*a*). Thus, delayed punishment did not influence reward preference as substantially as immediate punishment, indicating that rats discount the negative value of delayed punishment.

Rats demonstrated a significant decrease in locomotion during the delay after selection of the punished lever compared with the safe lever (*t*_(16)_ = −3.85, *p* = 0.001; Fig. 2*c*). This suggested that subjects were aware that delayed punishment was impending after reward delivery. There was no difference in latency to decide between the punished or safe reward trials (*t*_(16)_ = −0.45, *p* = 0.66).

### Sex differences in delayed punishment discounting

**Figure 3.**
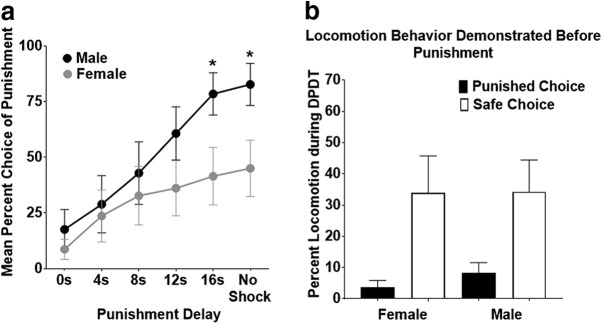
Sex differences in delayed punishment discounting. ***a***, Mean percentage of male versus female selection of the punished lever through blocks 1–6 of DPDT. Males discounted punishment significantly more than females as delay increased. Asterisks denote significant differences between sexes at individual time points. ***b***, Both females and males showed reduced locomotion during the delay preceding punishment compared with unpunished trials. Data displayed as mean ± SEM

A two-way mixed ANOVA was conducted to assess the impact of sex across the six different punishment latency blocks. While there was no main effect of sex (*F*_(1,18)_ = 2.066, *p* = 0.168), there was a significant sex × block interaction (*F*_(2.327,41.879)_ = 3.090, *p* = 0.049; Fig. 3*a*). *Post hoc t* tests revealed that males and females did not differ in choice of the punished reward during the first four blocks of DPDT, but males chose this reward more than females during the 16 s delayed punishment and no punishment blocks (Table 1). Thus, males and females demonstrated comparable devaluation of the punished reward when the shocks were delivered immediately or with shorter delays, but males discounted punishment more with the longest delay. Additionally, there was a near significant effect of sex on slope between blocks 1 (0 delay) and 5 (16 s delay; *t*_(18)_ = −2.02, *p* = 0.06), with females showing a mean slope of 7.26, and males demonstrating a steeper mean slope of 13.03.

**Table 1.** Sex differences in percentage choice of the punished reward for each delay block

**Delay**	**Sex**	**Mean**	**SEM**	***t***	**df**	***p***
0 s	Male	17.611	8.98	−0.882	18	0.389
	Female	8.722	4.56			
4 s	Male	28.933	12.87	−0.303	18	0.766
	Female	23.677	11.644			
8 s	Male	42.933	14.081	−0.53	18	0.603
	Female	32.727	13.155			
12 s	Male	60.711	11.934	−1.428	18	0.17
	Female	36.122	12.414			
16 s	Male	78.514	9.491	−2.311	18	0.033*
	Female	41.501	12.905			
No	Male	82.755	9.446	−2.389	18	0.028*
shock	Female	45.042	12.65			

**Figure 4.**
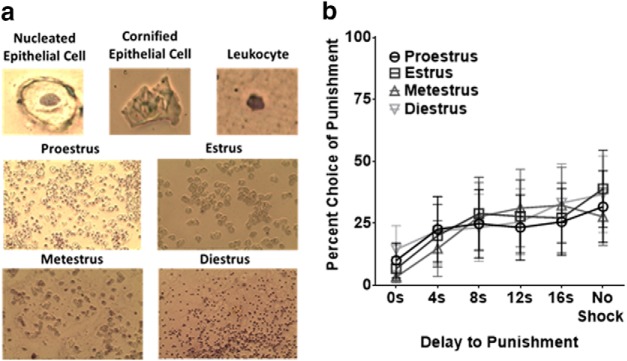
***a***, Appearance of nucleated epithelial cells, cornified epithelial cells, or leukocytes was used to determine stage of estrous cycle. ***b***, Female rats did not differ in percentage choice of the punished lever during the DPDT across proestrus, estrus, metestrus, and diestrus stages of the estrous cycle. Data depicted as mean ± SEM

A Levene’s test for equality of variances revealed no difference in variability between male and female rats in area under the DPDT curve (*F* = 0.003, *p* = 0.325). However, three females demonstrated complete avoidance of the large reward, even after removal of punishment during the final block, suggesting that females were more likely to use an avoidance-based strategy than males. A two-way sex × delay mixed ANOVA revealed that there were no significant sex differences in locomotion after choice of either reward (*F*_(1,15)_ = 0.124, *p* = 0.729; Fig. 3*b*), nor was there a sex × delay interaction (*F*_(1,15)_ = 0.604, *p* = 0.449). Thus, males and females demonstrated comparable reduced locomotion during the delay preceding punishment, suggesting that sex differences were not related to inability to anticipate impending shock. Finally, in female rats, there was no main effect of estrous cycle phase on punishment discounting (*F*_(1.531, 15.314)_ = 2.024, *p* = 0.172), or phase × delay interaction (*F*_(2.790,27.902)_ = 1.076, *p* = 0.372; Fig. 4), indicating that female rats performed similarly across all four stages of the estrous cycle.

### Cued DPDT

**Figure 5.**
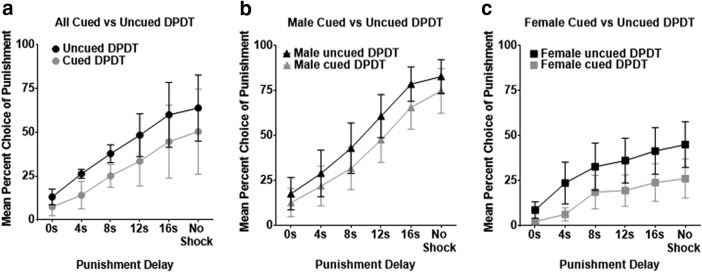
Cued DPDT. ***a***, Mean percentage of male versus female selection of the punished lever through blocks 1–6 of DPDT and cued DPDT. ***b***, There was no significant cue × sex interaction, indicating that addition of a cue light attenuated choice of the punished lever for (***b***) male and (***c***) female rats. Each marker represents mean ± SEM.

We next measured the influence of a visual cue bridging the gap between selection of the large reward lever and the delayed shock. Addition of this cue light significantly reduced choice of the punished reward (*F*_(1,17)_ = 16.012, *p* = 0.001; Fig. 5a). There was also no significant cue × block interaction (*F*_(2.158,36.691)_ = 1.242, *p* = 0.303), indicating that although the cue light reduced choice of the punished reward, it did not affect the shape of the discounting curve. The cue × sex interaction was not significant (*F*_(1,17)_ = 1.099, *p* = 0.309), indicating that presence of a cue exerted comparable effects on both male and female rats. Finally, there was no significant interaction between delay block and cue location (*F*_(1.957,29.354)_ = 1.602, *p* = 0.219), suggesting that cue location did not bias task performance. In summary, a punishment-predictive cue caused an overall reduction in choice of the punished reward without affecting the shape of the discounting curve across both sexes.

### Comparing DPDT with delay discounting

**Figure 6.**
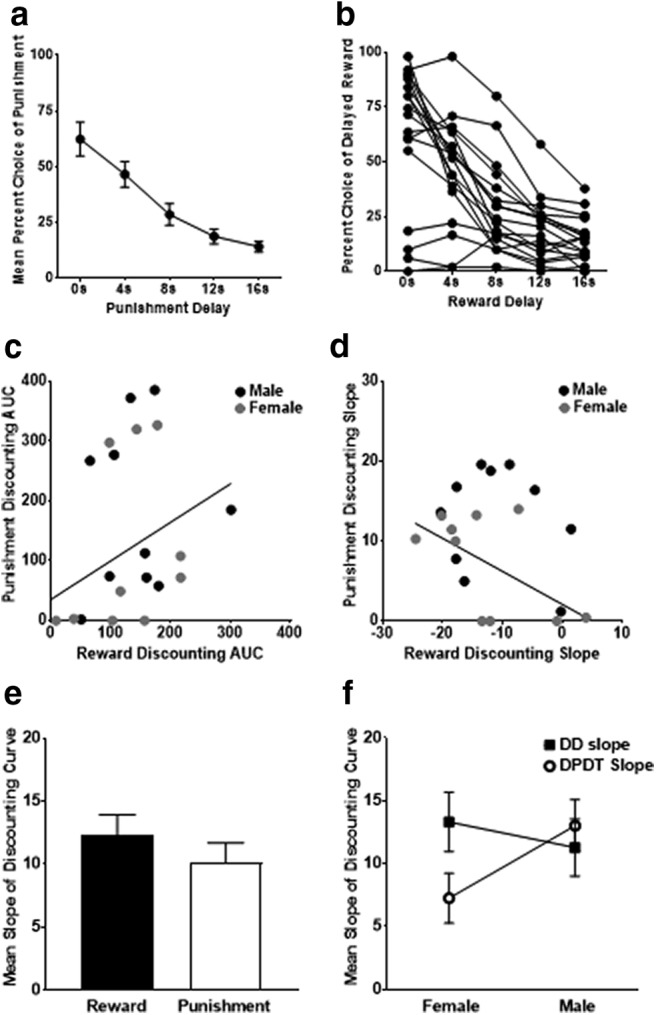
Comparing delayed reward and punishment discounting. ***a***, Rats shifted preference toward the immediate reward as the delay increased, indicative of delay discounting. ***b***, Individual differences in delay discounting, with each curve representing an individual rat. ***c***, AUC for discounting of delayed rewards was not significantly correlated with AUC during discounting of delayed punishment. ***d***, Slope of percentage choice of the punished reward lever during DPDT and percentage choice of the delayed reward lever during delay discounting were not correlated. ***e***, There was no differences in discounting curve slope between reward and punishment discounting. ***f***, When animals were separated by sex, females demonstrated a higher slope for delayed reward than delayed punishment, indicating more rapid discounting of delayed rewards versus punishments. No task difference was observed in males. ***a***, ***e***, and ***f*** display mean ± SEM; ***b***, ***c***, and ***d*** display individual scores.

Rats were trained in a small versus large, delayed reward discounting paradigm. Two females and one male were removed from data analyses because of enduring avoidance of the large reward. A mixed sex × delay ANOVA revealed a significant main effect of delay (*F*_(4,60)_ = 44.251, *p* < 0.001), indicating that rats discounted the large reward as a function of delay (Fig. 6*a*). There were no sex differences in reward choice during delay discounting (main effect of sex: *F*_(1,15)_ = 0.021, *p* = 0.888; sex × delay interaction: *F*_(1.760,26.399)_ = 1.213, *p* = 0.309).

We then analyzed the relationship between large¸ delayed reward choices in the delay discounting task with large, punished reward choice in DPDT. There was no correlation between AUCs for the DPDT and delay discounting tasks (*r* = −0.049, *n* = 17, *p* = 0.852; Fig. 6*c*); nor was there relationship between slopes of the discounting curves for each task (*r* = 0.160, *n* = 17, *p* = 0.539; Fig. 6*d*). When rats were separated into males and females, there were no correlations for either sex between task slopes (female: *r* = −0.207, *n* = 8, *p* = 0.623; male: *r* = −0.189, *n* = 9, *p* = 0.626; Table 2) or AUCs (female: *r* = 0.008, *n* = 8, *p* = 0.985; male: *r* = −0.098, *n* = 9, *p* = 0.802; Table 2). We also compared large reward choice between tasks at each individual delay and observed no significant correlations within all rats or either sex (for full statistics, see Table 2). Thus, delayed punishment discounting appears to be independent of delayed reward discounting.

**Table 2.** Bivariate correlations were conducted to compare slope, AUC, and mean percentage choice of the punished reward for each delay block between DPDT and the delay discounting task

**Measure**	***r* All rats**	***p* All rats**	***r* Female**	***p* Female**	***r* Male**	***p* Male**
Slope	−0.173	0.508	0.207	0.623	−0.237	0.539
AUC	−0.049	0.852	0.008	0.985	−0.098	0.802
Mean: 0 s	0.386	0.126	0.658	0.076	0.409	0.274
Mean: 4 s	−0.134	0.608	0.009	0.984	−0.199	0.608
Mean: 8 s	−0.269	0.296	−0.201	0.632	−0.347	0.361
Mean: 12 s	0	0.999	−0.021	0.961	−0.082	0.833
Mean: 16 s	0.068	0.796	−0.098	0.818	0.052	0.895

There were no significant correlations between tasks in any measure for all rats, nor for individual sexes.

To determine whether there was a difference in the rate of discounting between rewards and punishments, we compared the slopes of the respective discounting curves using a two-way mixed ANOVA. There was no difference in slope between discounting of reward and punishment (*F*_(1,18)_ = 1.572, *p* = 0.226; Fig. 6*e*); however, there was a sex × task interaction (*F*_(1,18)_ = 5.126, *p* = 0.036; Fig. 6*f*). Within-subjects *t* tests revealed that females had a greater discounting curve slope for rewards than punishments (*t*_(9)_ = 2.941, *p* = 0.016), whereas males showed no difference between task outcomes (*t*_(9)_ = −0.630, *p* = 0.544). Therefore, female (but not male) rats demonstrated more rapid discounting of delayed rewards than delayed punishments.

## Discussion

There is a wealth of research dissecting the cognitive and neuronal processes underlying delay discounting of rewards, yet there remains a paucity of knowledge on discounting of delayed punishment. To address this, we developed the DPDT. We observed that rats discount the negative value of delayed consequences relative to immediate consequences. Female rats discounted delayed punishment less than males, and this was not influenced by phase of the estrous cycle. Moreover, addition of a punishment predictive cue light decreased choice of delayed punishment for both sexes. Finally, there was no predictive relationship between the discounting of delayed rewards and punishments, although females selectively discounted reward at a faster rate than punishment.

### DPDT

The DPDT revealed that, on average, rats avoided a large reward associated with an immediate shock in favor of a small, safe reward. However, rats shifted preference toward the punished reward when the shock was delayed. This demonstrates that, like in humans (Murphy et al., 2001), rats discount delayed consequences relative to immediate consequences, and that this value transformation increases as a function of delay. The current task also revealed substantial individual differences in propensity to discount delayed punishment, which may be a promising avenue for future study.

One concern was that the delay caused the shift in preference via failure to associate actions with the delayed outcomes, rather than discounting of delayed consequences. To address this, we measured locomotion following choice of the large reward, and then compared this with a time-matched delay after choice of the small reward. Rats demonstrate freezing behavior during expectation of an aversive stimulus, quantified as near-complete lack of mobility and crouching posture (Fanselow, 1980). Although we did not directly assess freezing in the current study, cessation of normal locomotion is an integral portion of the freezing response; therefore, locomotion has utility as a proxy of freezing. Rats demonstrated a consistent reduction in locomotor activity during the pre-punishment delay, suggesting that rats were indeed aware of impending punishment, yet still discounted the negative value during reward choice.

Although delayed punishment discounting is relatively understudied, other studies have observed this factor in different animal models of decision-making. A recent study observed two-choice delayed punishment discounting during decision-making task in rodents (Rodríguez et al., 2018), but varied in numerous ways from the task at hand. The current study used different reward size and delay lengths, different ITI length, and included a punishment-free block as an additional control measure. Additionally, Rodríguez et al. (2018) used multi-colored cue lights to signify a change in delay time to foot shock, whereas our experiment was replicated in both un-cued and cued conditions. Another experiment involving rhesus monkeys used histamine as a punishment to reduce the potency of a cocaine reward, and observed that delaying the histamine infusions increased choice of the punished cocaine reinforcer (Woolverton et al., 2012). These variations of delayed punishment demonstrate that this phenomenon occurs across multiple species and rat strains.

### Sex differences in delayed punishment discounting

To our knowledge, this experiment was the first to report sex differences in delayed punishment discounting. We observed that male rats discounted delayed consequences more than females, as indicated by an increased shift in preference toward punishment-associated rewards when punishment was delayed. This did not appear to be a function of reduced overall sensitivity to punishment, as males and females demonstrated comparable avoidance of the punished reward when punishment was immediate, and also displayed comparable attenuation of movement during punishment anticipation. Another possible explanation for the sex difference is that females were becoming more satiated throughout the session, causing increased preference for the smaller reward compared with males. However, when rats acquired the initial one versus three pellet discrimination before introduction of foot shock, males and females showed equivalent preference for the larger reinforcer (data not shown), indicating that sex differences only manifested in the presence of delayed punishment.

These data expand on previous research that reported sex differences in response to punishment during reward seeking. Orsini et al. (2016) observed that female Long–Evans rats significantly preferred small, safe rewards over large rewards associated with risk of punishment relative to males, and that this was unrelated to body weight or reward motivation. Female rats also demonstrate increased latency to seek punished rewards relative to males (Chowdhury et al., 2019). Altered sensitivity to punishment in females is not limited to nociceptive stimuli, because female rats are more sensitive to reward loss than males (van den Bos et al., 2013).

Although the current study did not observe any statistical differences in variability between sexes, it is noteworthy that a subset of females (*n* = 3) but not males demonstrated complete avoidance of the punished reward. This female-specific avoidance behavior is congruent with Gruene et al. (2015), who observed that a subset of females performed an escape-like “darting” response to impending shock in lieu of the more passive freezing response traditionally observed in male subjects. Future studies will use larger samples of rats to more thoroughly assess potential differences in strategy selection in response to delayed punishment between male and female rats.

The estrous cycle mediates reward-seeking, cue sensitivity, and evoked dopamine release in females (Becker and Hu, 2008; Calipari et al., 2017; Johnson et al., 2019). Furthermore, there is evidence that women vary in discounting of delayed rewards during different phases of estrous (Hosseini-Kamkar and Morton, 2014). Thus, it was important to test whether estrous contributed to delayed punishment discounting. No difference was evident in DPDT during any of the four phases of estrous, consistent with estrous cycle playing no role in other punishment-related decision-making tasks (Orsini et al., 2016). Therefore, it is unlikely that hormonal fluctuations contribute to sex differences in delayed punishment discounting.

The majority of the seminal studies in behavioral neuroscience have been restricted to male subjects (Beery and Zucker, 2011). Unfortunately, this relegates many overarching theories of behavior to a singular male perspective, disregarding both the differences in brain structure/function and the discrepancies in vulnerability to disease between males and females (Becker and Hu, 2008; Grissom and Reyes, 2019; Shansky, 2019). The current study provides novel evidence that male behavior is not fully generalizable to females during economic decision-making, further underscoring the importance of evaluating behavior in both sexes to optimize treatment of maladaptive decision-making in psychopathology.

### Cue influence on delayed punishment discounting

In humans, providing reminders of delayed rewarding outcomes reduces both temporal discounting and vulnerability to substance use (Murphy and Dennhardt, 2016). Rodent models of reward-based delay discounting have demonstrated that introduction of a cue that bridges the gap between an action and the outcome affects task acquisition and sensitivity to drugs and brain region inactivation (Cardinal et al., 2000; Zeeb et al., 2010). Accordingly, we tested whether exposure to cues reminding subjects of impending negative outcomes affects delayed punishment discounting. We determined that the addition of a punishment-predictive cue reduced punishment discounting in both sexes, reflected as a shift in preference away from punished rewards preceded by delays. This finding reinforces the idea that environmental cues heavily influence temporal decision-making. Furthermore, this suggests that providing reminders of impending consequences may have utility for attenuating high levels of punishment discounting, which may be maladaptive in disorders characterized by pathologic reward seeking in the face of consequences.

### Delay discounting and delayed punishment discounting

Preference for immediate gratification, often referred to as delay discounting or impulsive choice, is prevalent in many mental disorders, including substance use disorder (Winstanley et al., 2004; Garavan and Hester, 2007; Brevers et al., 2012; Orsini et al., 2017). Although there is substantial literature in humans and animals investigating delayed rewards during decision-making, there is very little research investigating delayed consequences in animal models. Evidence from human studies suggest that the discounting of rewards and consequences are distinct cognitive processes that occur at different rates (Murphy et al., 2001). To further evaluate the independence of these constructs, we compared delayed punishment performance in this task with delayed reward performance in a traditional delay discounting task (modified from Simon et al., 2013). Both tasks used identical sets of delays (0, 4, 8, 12, 16 s) and comparable reward parameters (1 vs 3 pellets). As in humans, we observed no association between discounting of rewards or punishments. Interestingly, humans discount delayed rewards at a faster rate than delayed costs (Murphy et al., 2001). Although we did not observe this across all subjects, females showed a steeper discounting curve for rewards than punishments, being more likely to shift away from delayed rewards than to shift toward punishment at comparable delays. This sex-selective species difference may be a related to a critical difference in task outcomes: in the current study, the delayed consequence is a physical foot-shock, which is distinct from the delayed reward of pellets. In the aforementioned human study, both the reward and consequences manipulate the same outcome: either gaining or spending money, respectively (Murphy et al., 2001). This provides further evidence of sex differences in delayed outcome processing, which suggests differences in recruited neuronal circuitry or functional activity during decision-making.

Multiple brain regions are implicated in mediating time-discounting of rewards, including the orbitofrontal cortex, nucleus accumbens, and basolateral amygdala (Cardinal, 2006; Roesch et al., 2006; Bickel et al., 2014). The current research suggests that delay discounting does not occur at a comparable rate between rewards and punishment; thus, it is likely that the neuronal mechanisms underlying punishment discounting diverge from those involved with transformation of reward value. Further research is necessary to confirm whether there are distinct circuits involved across both reward and punishment delay discounting, or whether comparable circuits encode delayed outcomes independent of valence.

### Conclusion

We developed a delayed punishment discounting task that (1) demonstrates that rats discount the negative value of delayed consequences, (2) reveals novel sex differences in decision-making, (3) is modulated by punishment predictive cues, and (4) is independent of reward delay discounting. Delayed punishment discounting is a critical aspect of substance use disorder and other forms of pathology, during which future consequences are often undervalued in favor of immediate rewards. This task will enable assessment of the neurobiological mechanisms underlying this critical phenotype, as well as rigorous determination of the causal relationship between delayed punishment discounting and substance use.

## Synthesis

Reviewing Editor: Mark Baxter, Mount Sinai School of Medicine Decisions are customarily a result of the Reviewing Editor and the peer reviewers coming together and discussing their recommendations until a consensus is reached. When revisions are invited, a fact-based synthesis statement explaining their decision and outlining what is needed to prepare a revision will be listed below. The following reviewer(s) agreed to reveal their identity: Erin Calipari, Mihaela Iordanova, Michael McDannald.

The reviewers were very impressed with the novelty of the task, the importance of the question, and the quality of the writing of the manuscript. There were minor questions raised of issues that could be expanded or clarified -

1) The paper compares reward and punishment discounting using AUC and slopes. Could a less global measure could reveal some relationships? For example, taking the mean percent choice of punishment or delayed reward at each time point for each rat and correlating those.

2) The use of the term 'freezing' is an issue. Freezing itself is an overt behavioral response that can be measured. This is typically the absence of movement with a stiff rigid posture accompanied by hyperventilation/piloerection. As far as I can tell, freezing was never measured in these rats. Instead, locomotion was measured and used as a proxy for freezing. I think it is perfectly valid to use locomotion. Further, freezing and locomotion likely correlate, although not perfectly. However, if freezing was not measured, I do not think the term freezing should be mentioned in the results or figures (2 and 3 for example). Although it would be reasonable to discuss locomotion as a proxy for freezing in the discussion.

3) Three female rats were removed from analysis of locomotion for showing avoidance behavior during the pre-shock period. I understand why these subjects were removed, but I think the authors need to spend a little more time talking about about individual and sex differences with respect to this finding. They allude to individual differences somewhat already. These results would seem to touch on observations from other groups in which female rats show more active responses to impending threats (e.g. Gruene et al., eLife 2015)

Minor questions:

1. p. 8 line 198-199: was the left/right cue congruent with the left/right lever?

2. While after reading the manuscript it is clear that all tasks were ran in the same rats, it would be beneficial to make this clear from the start.

3. Line 347 missing article in front of decision-making task?

## Author Response

We thank the reviewers and editor for their thoughtful comments. Below is a point by point response to each comment (responses in italics). All changes and additions are highlighted in yellow in the manuscript; pages listed below correspond to the page numbers in the header of the document file.

1) The paper compares reward and punishment discounting using AUC and slopes. Could a less global measure could reveal some relationships? For example, taking the mean percent choice of punishment or delayed reward at each time point for each rat and correlating those.

We performed correlations between tasks at each individual delay (for example: 4 second delay to large reward compared to 4 second delay to punishment) and observed no significant correlations between tasks at any time point. We report these data in detail in a newly constructed Table 2 and page 13 of the results ('Comparing DPDT with Delay Discounting').

2) The use of the term 'freezing' is an issue. Freezing itself is an overt behavioral response that can be measured. This is typically the absence of movement with a stiff rigid posture accompanied by hyperventilation/piloerection. As far as I can tell, freezing was never measured in these rats. Instead, locomotion was measured and used as a proxy for freezing. I think it is perfectly valid to use locomotion. Further, freezing and locomotion likely correlate, although not perfectly. However, if freezing was not measured, I do not think the term freezing should be mentioned in the results or figures (2 and 3 for example). Although it would be reasonable to discuss locomotion as a proxy for freezing in the discussion.

We agree with this distinction and have adjusted the terminology accordingly throughout the paper. In addition, Figures 2 and 3 were corrected by either eliminating the term “freezing” or replacing the term with “locomotion”. Finally, we briefly discuss the ability to infer fear-related behavior from attenuated locomotion in the discussion ('Delayed Punishment Decision-Making Task', page 14).

3) Three female rats were removed from analysis of locomotion for showing avoidance behavior during the pre-shock period. I understand why these subjects were removed, but I think the authors need to spend a little more time talking about individual and sex differences with respect to this finding. They allude to individual differences somewhat already. These results would seem to touch on observations from other groups in which female rats show more active responses to impending threats (e.g. Gruene et al., eLife 2015)

We have expanded our presentation and interpretation of the individual differences in our female subjects. We report these data in the 'Sex Differences in Delayed Punishment Discounting' section in the results on page 11, clarifying that a small (n=3) subset of females displayed complete avoidance of the punished reward throughout the session, then reported non-significant results from a Levene's Test for Equality of Variances between sexes in area under the DPDT curve. We also address this finding and compare it with Gruene et al. in the corresponding sub-section of the discussion (page 16).

Minor questions:

1. p. 8 line 198-199: was the left/right cue congruent with the left/right lever?

The cue was located above the left/right retractable lever. This cue always corresponded spatially with the large reward + delayed shock lever. We now clarify this on page 8 in the methods section and added to the results section a mixed side X block ANOVA to show a lack of effect of cue location on task performance (page 12).

2. While after reading the manuscript it is clear that all tasks were ran in the same rats, it would be beneficial to make this clear from the start.

We clarified this point in the Subjects section of the Methods (page 5).

3. Line 347 missing article in front of decision-making task?

The Rodriguez et al., 2018 citation has been moved to the previous line to address this concern.
